# Spangled Hair in Siblings

**DOI:** 10.4103/0974-7753.51925

**Published:** 2009

**Authors:** Sundaram Murugusundram

**Affiliations:** Consultant Dermatologist/Secretary, The Hair Research Society of India, Chennai, India

Two north Indian siblings, an 11-year-old sister and an eight-year-old brother born to non-consanguineous parents and with a negative family history, presented with severe atopic eczema and abnormal scalp hair from the first year of life [Figure 1]. The hairs were dark, rough, short and straight like a broomstick with a spangled appearance under bright light [Figure 2]. There were no patches of alopecia. The hair density and tensile strength were clinically normal.

**Figure 1:**
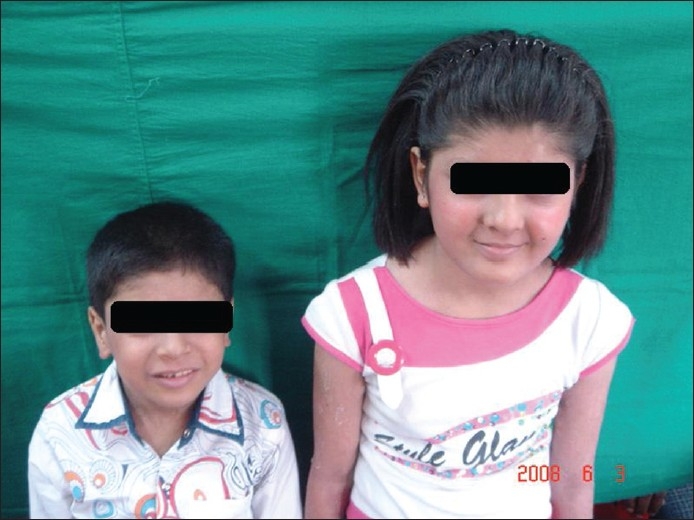
Siblings with abnormal scalp hair

**Figure 2:**
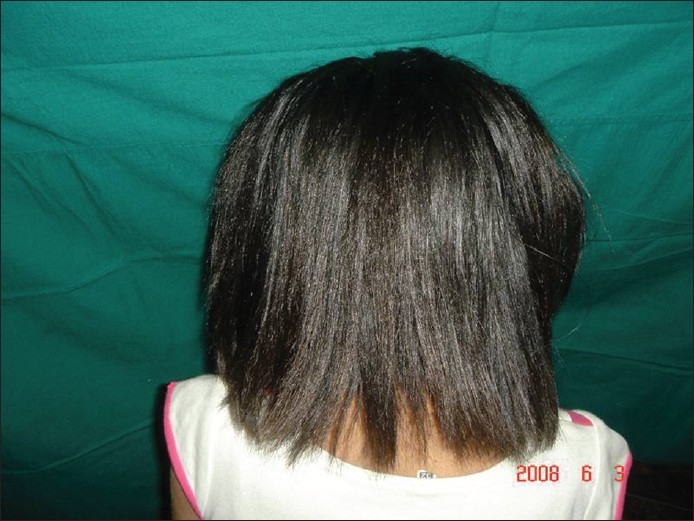
Spangled appearance with reflected light

Epiluminescence microscopy and light microscopy of the hair shafts was normal. Polarized microscopy showed alternating dark and light bands arranged parallel to the long axis of the hair shaft [Figure 3].

**Figure 3:**
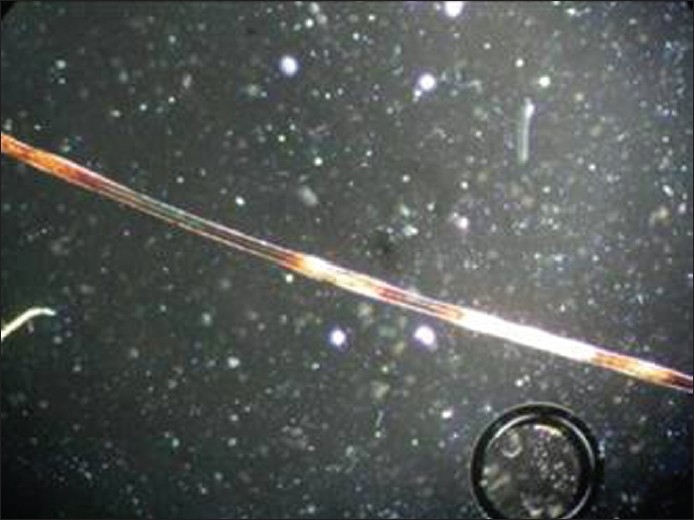
Alternate parallel banding under polarized microscopy

## WHAT IS YOUR DIAGNOSIS?

## DIAGNOSIS: PILI ANNULATI

The spangled appearance of hair under reflected light with alternate parallel banding under polarized microscopy favors the diagnosis of pili annulati. Association with atopy has not been reported in the literature according to my knowledge.

## DISCUSSION

Pili annulati is an extremely rare non-fragile hair shaft disorder characterized by banding leading to spangled hair. It is an autosomal dominant condition with a recently identified genetic locus on Chromosome 12.[1] Although it is clinically detectable in blond or lightly pigmented hair as speckled banding, it is only an incidental finding because of the non-fragility of hair.[2] It is difficult to diagnose this condition in dark hair since the banding is obscured by the pigment. The characteristic spangled appearance of hair with reflected light is the only clue in dark hair.

An inherent defect in the hair shaft leads to formation of air-filled cavities within the cortex that lie parallel to the long axis of the hair.[3] These are seen in the polarized microscopy as alternating light and dark bands. Examination of hair under electron microscopy shows cobble-stoning of the cuticle and air-filled cavities within the cortical cells and between the keratin macro fibrils.

Pili annulati has to be differentiated from pseudo pili annulati which is sometimes the feature of normal hair due to an optical illusion caused by partial twisting. Pili annulati differs from trichothiodystrophy which shows alternating light and dark bands arranged perpendicular to the long axis of the hair shaft resembling tiger's tail [Table 1].

**Table 1:** Trichothiodystrophy vs Pili annulati

Trichothiodystrophy	Pili annulati
Autosomal recessivet	Autosomal dominant
Faulty repair of DNA and low cysteine	Air-filled cavities within the cortex
Persistent alopecia of the scalp, eyebrows	No alopecia but spangled hair
Fragile hair	Non-fragile hair
Perpendicular banding – 'Tiger Tail'	Parallel banding
Many associated defects	Usually none. Rarely a few

Pili annulati is rarely associated with woolly hair, anhidrotic ectodermal dysplasia,[4] blue nevi [5] and alopecia areata.[6]Association with atopy is extremely rare. Since atopic children might have various hair shaft disorders it is worth studying a large population of atopic children for hair shaft abnormalities.

Pili annulati does not resolve spontaneously or improve with age. Since hairs are non-fragile no treatment is required.
